# Editorial: Respiratory Virus Infection: Recent Advances

**DOI:** 10.3389/fmed.2020.00257

**Published:** 2020-06-09

**Authors:** Ville Peltola, Shin-Ru Shih, Kelvin Kai-Wang To

**Affiliations:** ^1^Department of Paediatrics and Adolescent Medicine, Turku University Hospital and University of Turku, Turku, Finland; ^2^Research Center for Emerging Viral Infections, Chang Gung University, Taoyuan, Taiwan; ^3^Department of Microbiology, Li Ka Shing Faculty of Medicine, The University of Hong Kong, Pokfulam, Hong Kong

**Keywords:** respiratory virus, influenza, epidemiology, pathogenesis, therapeutics, vaccines

Respiratory virus infections cause an enormous disease burden both in children and in adults in all parts of the world. Influenza A and B viruses and respiratory syncytial virus (RSV) are responsible for the highest numbers of deaths and hospitalizations but other respiratory viruses, including rhinovirus, parainfluenza viruses, adenovirus, human bocavirus and coronaviruses, are also currently known to cause severe diseases in addition to mild upper respiratory tract infections. Furthermore, respiratory viruses of animal origin keep on emerging as novel human pathogens with epidemic or pandemic potential. Such viruses include avian influenza viruses and the Middle East Respiratory Syndrome (MERS) and Severe Acute Respiratory Syndrome (SARS) coronaviruses.

Despite the high clinical importance of respiratory viruses, the mechanisms of pathogenicity, transmission and evolution are only partially known, and most respiratory virus infections lack effective treatment or prevention modalities. With this Research Topic we aimed to focus on these knowledge gaps. We sought to collect original research articles and focused reviews from leading experts all over the world on respiratory virus epidemiology, pathogenesis, diagnostics, therapeutics and vaccines. We acknowledge all authors who submitted high-quality manuscripts. We accepted nine papers including six original research articles and three reviews. Influenza viruses are well-presented in the Topic by five papers. Two articles focus on RSV and one each on rhinovirus and MERS coronavirus.

Seasonal influenza A and B virus epidemics globally cause up to 600,000 deaths per year. Influenza A viruses have an animal reservoir in avian species, which forms the basis for a permanent threat of pandemics. Vaccines and specific antiviral drugs against influenza are available but they have limitations including variable vaccine efficacies. McAuley et al. review the current knowledge on the structure of the influenza virus neuraminidase and link the structural features of this surface glycoprotein with the multiple functions of neuraminidase in virus infection and fitness. Guo et al. studied the genetic changes that occur in avian influenza A H5 viruses when they infect humans. Identification of the important steps in adaptation of these viruses to humans is needed for evaluating the pandemic risk related to avian influenza viruses.

National reporting of clinical microbiology results is an important part of surveillance for respiratory virus epidemics. Clinical microbiology services have recently been consolidated in many countries to a limited number of central laboratories. Van den Wijngaert et al. studied the effects of such laboratory merging on influenza surveillance in Belgium. They report benefits of the integration of whole genome sequencing of influenza viruses performed in centralized clinical microbiology laboratories into the public health surveillance system.

Neuraminidase inhibitors are widely used for treatment (and sometimes prevention) of influenza infection, and they have demonstrated efficacy. However, new drugs are needed to reach better treatment results in vulnerable patient groups particularly in the scenario of developing antiviral resistance. Several new antiviral drugs against influenza have been developed or are under development, including polymerase inhibitors and monoclonal antibodies. These new drugs are reviewed in the paper by Principi et al..

Vaccines are the cornerstone in protection against influenza. However, currently used influenza vaccines have limitations and the process of production in eggs is vulnerable. Yamada et al. report preclinical results on the efficacy of a recombinant hemagglutinin protein vaccine candidate produced in human Expi293F cell culture. Interestingly, recombinant hemagglutinin protein of H1N1pdm09 virus protected mice not only against H1N1pdm09 virus but also against avian H5N1 influenza virus.

RSV occurs in yearly epidemics that cause high number of severe lower respiratory tract infections in young infants and also in the elderly. There is variation in the timing of RSV epidemics between geographic areas. Recognition of the onset of the epidemic is important in order to accurately start adequate control measures, particularly passive immunization of high-risk infants by monoclonal antibodies such as palivizumab. Yamagami et al. developed a mathematical model that was able to detect the onset of RSV season in different parts of Japan with various climate conditions. Another interesting study on RSV by Hong et al. focused on enhancing antiviral activity of alveolar macrophages by administration of *Bacillus subtilis* spores in a mouse model. Their findings may help in development of novel RSV therapeutics in the future.

Rhinovirus is frequently detected in children with pneumonia, but its clinical significance remains unclear. Hartiala et al. compared rhinovirus-positive and rhinovirus-negative children hospitalized with community-acquired pneumonia. They found that the clinical picture was rather similar in both groups. Premature birth associated with rhinovirus-positive pneumonia. More research is needed on the pathogenic role of rhinovirus in pneumonia.

Skariyachan et al. reviewed the pathogenesis, animal models and therapeutics for MERS coronavirus infections. This review is timely not only for the continuing identifications of human infections by MERS coronavirus in the Middle East but also because of the potential of other novel coronaviruses to jump from animals to humans. Most recently, a novel beta coronavirus has led to an outbreak of respiratory tract infection in Wuhan, China.

We believe that the articles published in this Research Topic provide important new knowledge on respiratory virus infections. They identify areas of lacking knowledge that need to be addressed by future research. We hope that this collection of articles is beneficial for the readers and advances the field.

## Author Contributions

VP drafted the editorial and all authors revised it.

## Conflict of Interest

The authors declare that the research was conducted in the absence of any commercial or financial relationships that could be construed as a potential conflict of interest.

